# Vectors, Hosts, and the Possible Risk Factors Associated with Severe Fever with Thrombocytopenia Syndrome

**DOI:** 10.1155/2021/8518189

**Published:** 2021-11-03

**Authors:** Jin-Na Wang, Tian-Qi Li, Qin-Mei Liu, Yu-Yan Wu, Ming-Yu Luo, Zhen-Yu Gong

**Affiliations:** Zhejiang Provincial Center for Disease Control and Prevention, 3399 Binsheng Road, Binjiang District, Hangzhou 310051, China

## Abstract

Severe fever with thrombocytopenia syndrome (SFTS) is a disease caused by infection with the SFTS virus (SFTSV). SFTS has become a crucial public health concern because of the heavy burden, lack of vaccines, effective therapies, and high-fatality rate. Evidence suggests that SFTSV circulates between ticks and animals in nature and is transmitted to humans by tick bites. In particular, ticks have been implicated as vectors of SFTSV, where domestic or wild animals may play as the amplifying hosts. Many studies have identified antigens and antibodies against SFTSV in various animals such as sheep, goats, cattle, and rodents. Besides, person-to-person transmission through contact with blood or mucous of an infected person has also been reported. In this study, we reviewed the literature and summarized the vectors and hosts associated with SFTS and the possible risk factors.

## 1. Introduction

Severe fever with thrombocytopenia syndrome (SFTS) is an emerging infectious disease, caused by SFTS virus (SFTSV), a novel *Phlebovirus* in the Bunyaviridae family, which was first identified in China [1]. Since the first report in 2009, SFTS has attracted great public health attention in Asia, especially from China, Japan, and South Korea [2–4]. Besides, recently, a related study has also been reported in Vietnam [5]. The clinical manifestations of SFTS include fever, thrombocytopenia, gastrointestinal symptoms, hemorrhagic tendency, and multiple organ dysfunctions, with 5–14 days of incubation and a high initial case fatality rate of 30% [1, 6]. In total, 96.5% of the SFTS cases occurred from April to October, and in China, 93.3% of the cases were reported in middle-aged and elderly people with a spatially expanding trend [7]. Among them, farmers were the main high-risk population, and there were more cases in female than males [7]. Although the natural transmission mode of SFTSV among humans, hosts, and vectors remains unclear, increasing evidence has showed that ticks may be the transmission vector, while the domestic or wild animals may play as the amplifying hosts.

## 2. Main Text

Vectors, hosts, and the possible risk factors associated with SFTS.

### 2.1. Ticks

The infection rate of the ticks is varied in different species, developmental stages, genders, regions, or even the collected hosts. Studies showed the pooled infection rate of SFTSV in ticks was 0.08 [8], and the average minimum infection rate in larvae was 0.57%, nymphs 0.36%, males 0.90%, females 1.53%, fed ticks 18.2%, and unfed ticks 5.9% [9, 10]. In Table 1, more than 16 tick species collected from animals or vegetation were detected, with at least nine tick species including *Haemaphysalis longicornis* (*H*. *longicornis*), *H*. *flava*, *H*. *concinna*, *H*. *formosensis*, *H*. *hystricis*, *H*. *megaspinosa*, *Ixodes nipponensis* (*I*. *nipponensis*), *Amblyomma testudinarium* (*A*. *testudinarium*), and *Rhipicephalus microplus* (*R*. *microplus*) were found positive in SFTSV RNA detection [3, 9, 11, 15, 17–19]. The *Haemaphysalis* had attracted the most attention, and at least 6 species were test to be positive. The *H*. *longicornis* were suspected to be the major tick species in most areas, and the higher positive rate was 4.84% collected from animals in China [13] and 4.77% from vegetation in ROK [14]. There were also negative results of RNA detection in tick species, such as *H*. *japonica*, *H*. *yeni*, *H*. *campanulata*, *H*. *phasiana*, *I*. *turdus*, and *Dermacentor sinicus* (*D*. *sinicus*) [11, 13, 15, 16, 18]. Regarding the observed negative results, there were several possible explanations. First, due to the relatively small sizes and limitation of collection sites, the tick sample was not representative. Second, low positive rate of SFTSV in ticks and the limited viral loads may also be responsible for these negative results [22]. Tick bite has been considered to be the most important risk factor for SFTS, which was significantly associated with the SFTSV infection [17]. Ticks, especially the *H*. *longicornis*, which had higher density, wider distribution, and higher detection rate were suggested to be the primary reservoir and vector of SFTSV [23], with increasing supportive evidence. First, some SFTS patients have reported a history of tick bite, and the spatial and temporal distributions of SFTS human cases were consistent with the fluctuation of certain species of ticks in the endemic area [24–26]. Besides, the SFTSV-specific nucleotide sequences or virus isolated from ticks had high homology with those from the patients [27]. Second, the transmission feeding experiment found that *H*. *longicornis* fed on SFTSV-infected mice could acquire the virus and transstadially and transovarially transmit it to other developmental stages of ticks, and furthermore, the infected ticks could also transmit the virus to mice during feeding [28]. Besides, the transovarially and horizontally transmissions were also found in *I*. *sinensis* ticks [29]. Third, SFTSV RNA could be identified in larvae of ticks collected in vegetation without being blood fed, indicating the possibility of a vertical transmission and ticks as a putative reservoir host [30]. Except for ticks, mosquitoes and sandflies were also detected. In the first report of SFTSV, Yu et al. had already found that viral RNA was not detected in any of 5900 mosquitoes collected from the home environment of the patients [1]. Using experimental infections of mosquitoes with SFTSV, Liang et al. examined the role of mosquitoes in the transmission of the virus and did not detect viral replication in *Culex pipiens pallens*, *Aedes aegypti*, and *Anopheles sinensis* as revealed by the TaqMan quantitative real-time polymerase chain reaction (qRT-PCR) assay. Besides, the authors failed to isolate SFTSV from the Vero cells cultured with suspensions of SFTSV-infected mosquitoes [31]. Similarly, in another study, viral RNA was not detected in any tested mosquitoes, midges, and sandflies [11].

### 2.2. Domestic Animals

SFTSV is thought to circulate in an enzootic tick-vertebrate-tick cycle, yet the vertebrate hosts in nature have not been confirmed [30]. SFTS viral RNA was most commonly detected among domesticated animals, followed by ticks and humans [17]. One meta-analysis study found the pooled seroprevalence of anti-SFTSV antibodies was 45.70% in goats and sheep, 36.70% in cattle, 29.50% in dogs, 9.60% in chickens, 3.20% in rodents, and 3.20% in pigs, respectively [32]. In Table 2, SFTSV antibodies or RNA have been detected positive in more than ten kinds of domesticated animals, including dogs, goats, cattle, and pigs [4, 6, 11, 12, 17, 33, 39, 41, 42]. Among them, the highest antibody-positive rate was seen in cattle (97.92%) of China [36]. Additionally, high SFTSV antibody rates were also found in goats (74.77%), chickens (52.14%) [37], sheep (69.74%), dogs (68.18%), ducks (19.67%) [36], minks (8.43%) [6], cats (1.92%) [38], pigs (4.66%), and geese (1.67%) [35]. Besides, in ROK and China, SFTSV RNA was also found to be positive in cats (33.08%) [42], cattle (2.08%) [36], goats (1.93%) [39], pigs (1.67%) [41], and dogs (0.88%) [38]. Domestic animals, especially the companion animals, could increase the epidemic threat of SFTS due to the close interaction with humans [43]. Ticks predominantly feed on farm animals including goats, sheep, cattle, chicken, and also on companion animals such as dogs and cats [33, 34]. The domestic animals could acquire SFTS and play as the amplifying hosts and viral spread [24, 44]. Except for the vector of ticks, domestic animals could transmit SFTS in another way. High levels of SFTSV RNA loads could be detected in serum, eye swab, saliva, rectal swab, and urine of the domestic animals, indicating a risk of direct human infection from SFTS-infected animals [45]. A patient could acquire SFTS by blood splash when removed or burst ticks from the dogs with his bare hands [46]. Three SFTS patients had direct contact with body fluids of ill companion animals, and two SFTS patients had direct contact with saliva of an ill feral cat or pet dog, without a history of tick bite [47]. A woman was infected with SFTS two days after the bites from a sick cat [48]. From this perspective, the direct contact with body fluids of ill companion animals should be avoided. However, there were no direct evidence to support this viral transmission route. An experimental infection study in goats showed that the infected animals could not shed virus to the control animals through the respiratory or digestive tract route, and however, the control animals were infected after ticks' infestation [49]. Thus, more evidence is still needed to prove that whether animals could transmit SFTSV directly.

### 2.3. Wild Animals

Among the wild animals, the migratory birds probably played a role in the transmission of the SFTSV. The migratory wild birds could carry the virus, either through their own infection or carrying attached ticks infected to distant regions via migratory flyways [30, 50, 51]. Besides, many other species of small wild animals infected with SFTSV may serve as the natural amplifying hosts. In Table 3, the SFTSV antibodies or RNA have been detected in more than 16 kinds of wild animals, namely, rodents, Asian house shrews, hedgehogs, water deer, wild deer, wild boars, Siberian roe deer, gorals, raccoon dogs, carrion crow, yellow weasels, hares, rock pigeons, pheasants, turtledoves, and mongooses [4, 23, 35, 36, 38, 40, 52–55]. The highest SFTSV antibody positive rate was seen in yellow weasels (91.11%) detected in China [36]. Besides, a high antibody positive rate was also found in hares (63.01%), pheasants (42.86%), hedgehogs (40.00%), rock pigeons (34.78%) [36], wild deer (25.00%), wild boars (25.00%) [4], and water deer (23.81%) [23]. It is noteworthy that the positive rate varied in the same kind animals in different studies. Up to now, no positive results have been found in Siberian roe deer, gorals, raccoon dogs, and carrion crows [23]. The highest SFTSV RNA-positive results were seen in water deer (4.76%) [23], wild boars (5.21%) [55] yellow weasels (2.22%), hares (2.74%) [36], Asian house shrews (2.6%), and rodents (0.7%) [52]. SFTSV has also been detected in the ticks collected from wild animals such as lizards and snakes, indicating that they may be the potential hosts of SFTSV [56]. Besides, the rhesus macaques could be infected with SFTS similar to human, which may be used to develop adequate animal models [57].

### 2.4. Risky Behavior

SFTS was mostly endemic in rural areas [58]. A case-control study conducted in Henan, Hubei, and Shandong provinces of China suggested that the cases were more likely to be farmers than controls (88.8% vs. 58.7%) [26]. Presence of weeds and shrubs in working areas or around the house, as well as domestic animals illness or death two weeks prior to disease onset, tick bites, tea-picking, grazing, farming, raising domestic animals, mowing, presence of rats, or contact with wild animals were risk factors for SFTSV infection [27, 35, 59, 60]. Farming activities may be one index of the SFTS etiologic diagnosis due to the facts that the farming activities around weeds and shrubs increase the exposure opportunities for ticks [27]. Raising animals may also increase the risk of tick contact and SFTS infection [44], and there were significant differences in the history of tick bite and breeding domestic animals between cases and controls [24]. Other epidemiological characteristics such as the seasonality and meteorological factors substantially differed across the affected area. Liu et al. found eight predictors were significantly associated with the occurrence of SFTSV, including temperature, rainfall, relative humidity, sunshine hours, elevation, distribution of *H*. *longicornis* ticks, cattle density, and coverage of forest [2]. The SFTS epidemic curve revealed that 83.7% cases occurred between May and July [58], which is consistent with local agricultural activities and the seasonal abundance of ticks. One study showed that the risk of SFTS was high in geographic areas where the farmland area begins to diminish and at midlevel altitudes [61].

### 2.5. Age and Sex

Age is an important determinant for SFTS morbidity and mortality, and elder age and high viral load were significantly associated with fatal clinical outcomes [62]. Li et al. found the mean age of seropositive farmers was 56.5 years and seroprevalence increased gradually with age [35]. One study analyzed 7721 laboratory-confirmed SFTS cases in China from 2010 to 2018 and found that residents more than 60 years old in rural areas with crop fields and tea farms were at an increased risk of SFTS [50]. Sex was another associated factor of SFTS, with the findings that the SFTSV antibodies were more prevalent in males than in females (1.87% vs. 0.71%, *P* < 0.01) [35]. A study with 44 fatal SFTS cases showed that 16 (36.4%) were female and 28 (63.6%) were male [58]. As for the significant difference between age and sex, several hypotheses have been proposed. First, the decreased immune function and the more probability of comorbidities with chronic diseases in the elders may aggravate the SFTS fatality. Second, due to the fact that many young adults have moved to cities for better job opportunities, the elders from rural areas, especially the males, then have to assume the agricultural activities, which would make them more likely to be exposed to tick bites [2]. Besides, genetic susceptibility in the sexes may also be a reason for the difference in SFTSV infection and fatal outcome [63].

### 2.6. Person-to-Person Transmission

Person-to-person transmission of SFTSV has been proposed through close contact with blood or body secretions of infected patients [64]. The incubation period of the secondary patients was 10.0 days. The secondary attack rate (SAR) was 1.72–55.00%, and the average basic reproductive number (R0) was 0.13 (95% CI: 0.11–0.16) [65]. Risk factors assessment of the person-to-person transmission revealed that the major exposure factors may be blood contact without personal protection equipment (PPE) [66]. SFTSV could be transmitted through contact with patients' cadaveric blood or bloody secretions by the unprotected skin or mucosa, indicating that SFTSV-infected blood may remain infectious for a long time, even after the death of patients [67]. A person was 25 times more likely to be infected with SFTS when contacted with blood [68], and the frequency of blood contact was found to be associated with the disease risk in a dose-response pattern [69]. Besides, contact with bloody secretions or feces [70], aerosol [71], and various body fluids including urine and tracheal aspirate contained SFTS virus [72] of the index patient without PPE may be potential sources of SFTS transmission.

### 2.7. Nosocomial Transmission

After investigating the possible contamination of hospital air and surfaces with SFTSV transmission, Ryu et al. found that the swab samples obtained from stethoscopes, doorknobs, and bed guardrails were positive, which were also seen in television monitors and sink tables despite being remote from the patients [73]. These findings indicated that the extensive environmental contamination of SFTSV occurred in rooms of critically ill patients with SFTS. Kim et al. found that of the 27 healthcare workers (HCWs) who contacted with a fatally ill patient with SFTS, four involved in cardiopulmonary resuscitation complained of fever and were diagnosed with SFTS [74]. Similarly, another study reported that among 25 HCWs who had direct contact with the index patient, five who had contact to blood or bloody respiratory secretions of the index patient without adequate use of PPE were infected with SFTS [75]. SFTS cases could also occur in hospital in other ways, such as needle-stick injury in medical activities [76] and handling sick animals [77]. One study examining the seroprevalence of SFTSV infection in veterinarian staff members found that 3 of 71 (4.2%) were seropositive for SFTSV-specific antibodies [42]. Consequently, the strict adherence to routine blood and body fluid precautions is necessary when HCWs have contact with any patient. Furthermore, universal precaution and full PPE is highly recommended when there are signs of bleeding.

## 3. Conclusions

In conclusion, there is increasing evidence suggesting that ticks may be the primary vector of SFTSV, while domestic or wild animals play as the reservoir of the virus. Raising animals, as well as farming and grazing activities, especially among people characterized by elder age and male may be risk factors for SFTSV infection. Person-to-person transmission and nosocomial transmission have also been identified. Furthermore, the main host animals and mechanisms of transmissions need more deep-in research.

## Figures and Tables

**Table 1:** The results of SFTSV RNA detection in ticks.

Tick species	Sources	Positive rate (%)	Positive pools	Test pools	Country
*H*. *longicornis*	Animal	0.00	0	2	China [11]
	Animal	0.99	3	303	China [12]
	Animal	0.46	55	11856	ROK [9]
	Animal	4.84	65	1342	China [13]
	Vegetation	1.30	6	461	China [13]
	Vegetation	4.77	48	1006	ROK [14]
	Vegetation	0.06	11	17570	ROK [15]
	Vegetation	0.00	0	732	ROK [16]
	Animal and vegetation	4.40	8	182	China [17]
	Vegetation	0.00	0	3	Japan [18]
*H*. *flava*	Vegetation	1.15	5	436	ROK [14]
	Vegetation	0.24	8	3317	ROK [15]
	Vegetation	0.00	0	62	ROK [16]
	Vegetation	12.50	1	8	Japan [18]
*H*. *concinna*	Animal	21.25	17	80	China [11]
*H*. *japonica*	Animal	0.00	0	3	China [11]
*H*. *yeni*	Animal	0.00	0	1	China [11]
*H*. *campanulata*	Animal	0.00	0	9	China [13]
*H*. *phasiana*	Vegetation	0.00	0	8	ROK [15]
*H*. *formosensis*	Vegetation	23.53	4	17	Japan [18]
*H*. *hystricis*	Vegetation	50.00	2	4	Japan [18]
*H*. *kitaokai*	Vegetation	0.00	0	7	Japan [18]
*H*. *megaspinosa*	Vegetation	33.33	1	3	Japan [18]
*I*. *nipponensis*	Vegetation	0.00	0	23	ROK [14]
	Vegetation	0.40	1	249	ROK [15]
	Vegetation	0.00	0	2	ROK [16]
*I*. *turdus*	Vegetation	0.00	0	3	ROK [15]
*A*. *testudinarium*	Vegetation	20.00	1	5	ROK [14]
	Vegetation	0.00	0	11	ROK [15]
	Vegetation	50.00	2	4	Japan [18]
*R*. *microplus*	Animal	8.00	4	50	China [13]
*D*. *sinicus*	Animal	0.00	0	5	China [13]
Ticks unclassified	Animal and vegetation	3.00	33	1095	China [19]
	Vegetation	0.11	9	8313	ROK [3]
	Vegetation	4.81	9	187	ROK [20]
	Vegetation	2.12	63	2973	ROK [21]

**Table 2:** The results of SFTSV antibody detection and RNA detection in domestic animals.

Animals	Antibody positive rate (%)	Positive pools	Test pools	RNA positive rate (%)	Positive pools	Test pools	Country
Dogs	—	—	—	0.23	1	426	ROK [33]
	13.85	59	426	—	—	—	ROK [34]
	—	1	1	—	1	1	ROK [5]
	15.00	—	—	—	—	—	China [11]
	7.40	23	311	—	—	—	China [35]
	68.18	30	44	—	—	—	China [36]
	35.85	19	53	—	—	—	China [37]
	9.65	11	114	0.88	1	114	Japan [38]
	10.64	5	47	—	—	—	Japan [4]
Goats	66.70	—	—	—	—	—	China [11]
	66.79	185	277	—	—	—	China [35]
	9.09	2	22	—	—	—	China [17]
	74.77	83	111	—	—	—	China [37]
	14.49	30	207	1.93	4	207	ROK [39]
	48.29	521	1079	—	—	—	China [12]
	0.00	0	352	—	—	—	Japan [40]
Cattle	13.20	—	—	—	—	—	China [11]
	28.18	62	220	—	—	—	China [35]
	26.32	5	19	—	—	—	China [17]
	97.92	47	48	2.08	1	48	China [36]
	57.07	105	184	—	—	—	China [37]
Pigs	0.00	—	—	—	—	—	China [11]
	4.66	17	365	—	—	—	China [35]
	—	—	—	1.67	4	240	ROK [41]
	3.17	2	63	—	—	—	China [36]
	0.00	0	176	—	—	—	China [37]
Chickens	1.20	6	501	—	—	—	China [35]
	0.00	0	9	—	—	—	China [17]
	23.17	19	82	—	—	—	China [36]
	52.14	61	117	—	—	—	China [37]
Cats	—	—	—	0.47	1	215	ROK [33]
	—	—	—	33.08	44	133	Japan [42]
	1.92	2	104	0.00	0	104	Japan [38]
	0.00	0	386	—	—	—	Japan [40]
Geese	1.67	2	120	—	—	—	China [35]
Sheep	69.74	53	76	—	—	—	China [36]
Ducks	19.67	12	61	—	—	—	China [36]
Minks	8.43	15	178	—	—	—	China [6]

**Table 3:** The results of SFTSV antibody detection and RNA detection in wild animals.

Animals	Antibody positive rate (%)	Positive pools	Test pools	RNA positive rate (%)	Positive pools	Test pools	Country
Rodents	4.36	38	872	—	—	—	China [35]
	7.69	4	52	—	—	—	China [36]
	0.9	6	666	0.7	3	440	China [52]
Asian house shrews	4.5	4	89	2.6	2	77	China [52]
Hedgehogs	2.67	2	75	—	—	—	China [35]
	40.00	6	15	—	—	—	China [36]
Water deer	23.81	5	21	4.76	1	21	ROK [23]
	—	—	—	2.33	3	129	ROK [53]
Wild deer	0.00	0	314	—	—	—	Japan [54]
	25.00	5	20	—	—	—	Japan [4]
	34.58	37	107	0.00	0	107	Japan [38]
Wild boars	1.85	1	54	3.70	2	54	ROK [23]
	18.95	36	190	—	—	—	Japan [36]
	25.00	10	40	—	—	—	Japan [4]
	0.00	0	82	—	—	—	Japan [40]
	14.32	110	768	5.21	40	768	ROK [55]
	53.92	55	102	0.98	1	102	Japan [38]
Siberian roe deer	0.00	0	3	—	—	—	ROK [23]
Gorals	0.00	0	5	—	—	—	ROK [23]
Raccoon dogs	0.00	0	7	—	—	—	ROK [23]
Carrion crows	0.00	0	1	—	—	—	ROK [23]
Yellow weasels	91.11	41	45	2.22	1	45	China [36]
Hares	63.01	46	73	2.74	2	73	China [36]
Rock pigeons	34.78	8	23	—	—	—	China [36]
Pheasants	42.86	6	14	—	—	—	China [36]
Turtledoves	14.29	1	7	—	—	—	China [36]
Mongooses	4.19	9	215	—	—	—	Japan [40]

## Data Availability

The data used to support the findings of this study are included within the article.
